# The HIFIA/LINC02913/IGF1R axis promotes the cell function of adipose-derived mesenchymal stem cells under hypoxia via activating the PI3K/AKT pathway

**DOI:** 10.1186/s12967-023-04581-x

**Published:** 2023-10-17

**Authors:** Xiang Xiong, Liqin Yuan, Kai Yang, Xiancheng Wang

**Affiliations:** 1 Department of Plastic and Aesthetic (Burn) Surgery, The Second Xiangya Hospital, Central South University, Changsha, 410011 China; 2 Department of General Surgery, The Second Xiangya Hospital, Central South University, Changsha, 410011 China

**Keywords:** Hypoxia, Hypoxia-inducible factor-1alpha, LINC02913, IGF1R, PI3K/AKT pathway, Adipose-derived mesenchymal stem cells

## Abstract

**Objective:**

Promoting angiogenesis is crucial for tissue repair. Adipose-derived mesenchymal stem cells (ADSCs) are endowed with the ability of paracrine secretion of various angiogenic cytokines and the differentiation potential into endothelium-like cells to directly participate in angiogenesis. ADSCs are key seed cells for promoting angiogenesis in regenerative medicine and tissue engineering. This study aimed to explore the role and mechanism of C9orf106 (LINC02913) in the angiogenesis of ADSCs.

**Methods:**

The microarray dataset GSE12884 was analyzed to identify the differentially expressed lncRNAs in ADSCs under normoxia and hypoxia. The expression of the key genes was detected using qRT-PCR, western blot assay (western blot), and immunofluorescence (IF) staining. The adipogenic ability and tube formation ability of ADSCs was detected using oil red O staining and tube formation assay, respectively. The regulatory relationship between hypoxia-inducible factor-1alpha (HIF1A) and LINC02913 was verified using chromatin immunoprecipitation (ChIP) assay and dual-luciferase reporter gene assay. A skin wound healing nude mice model was established. Hematoxylin and eosin (H&E) staining was applied to detect pathological skin damage. Immunohistochemistry (IHC) staining was used to determine the level of CD31 in skin tissues.

**Results:**

LINC02913 expression was decreased in ADSCs under hypoxia; LINC02913 overexpression inhibited the proliferation, adipogenic ability, endothelial differentiation ability, and tube formation ability of ADSCs. ChIP assay and dual-luciferase reporter gene assay results showed that HIF1A could directly bind to the LINC02913 promoter region to inhibit its transcription. Through RNAact prediction and analysis of the correlation with LINC02913 expression, it was found that IGF1R may directly interact with LINCO02913. The HIF1A/LINC02913/IGF1R axis could activate the PI3K/AKT pathway to promote the biological function of ADSCs. Hypoxia-ADSCs significantly promoted vascularization in the wounded skin. The regulatory effect of LINC02913/IGF1R axis on hypoxia-ADSCs treated skin wound healing were verified.

**Conclusion:**

The HIF1A/LINC02913/IGF1R axis promoted the proliferation, adipogenic ability, and tube formation ability of ADSCs under hypoxia via activating the PI3K/AKT pathway.

**Supplementary Information:**

The online version contains supplementary material available at 10.1186/s12967-023-04581-x.

## Introduction

Angiogenesis is crucial in regenerative medicine. However, due to the difficulty of separating a large number of autologous blood vessels, there are limitations for applying vascular endothelial cells in angiogenesis. Recently, it has been shown that adipose-derived mesenchymal stem cells (ADSCs) are expected seed cells for promoting angiogenesis in regenerative medicine and tissue engineering, with their potent ability of paracrine secretion of various proangiogenic cytokines and differentiation potential into endothelium-like cells to directly affect angiogenesis [1, 2].

Long non-coding RNAs (lncRNAs), a class of non-coding RNAs longer than 200 nucleotides, lack specific complete open reading frames and protein-coding functions [3]. LncRNAs could regulate gene expression at various levels, involving multiple cellular functions, such as chromosome modification, transcriptional interference, and transcriptional activation [4]. Additionally, lncRNAs take part in a variety of biological processes, including cell migration, death, and angiogenesis. Recent years have seen an increase in research that show how lncRNAs regulate angiogenesis [5–7]. In addition, it has been shown that lncRNAs may be important for ADSC differentiation. For instance, by targeting miR-145-5p/Krüppel-like factor 4, the lncRNA maternally expressed 3 (MEG3) can cause mouse-derived ADSCs to differentiate into endothelial cells (KLF4) [8]. LINC02913, a newly identified lncRNA in recent years, has been found to be a promising biomarker and potential target in multiple diseases, such as ischemic stroke [9] and aortic dissection [10]. Nevertheless, the underlying role of LINC02913 in the differentiation and angiogenesis of ADSCs remains largely unknown.

Hypoxia-inducible factor-1alpha (HIF1A) is the main regulator that drives transcriptional response under hypoxia [11]. As a transcription factor, HIF1A is implicated in various biological activities, such as angiogenesis, glucose metabolism, cell proliferation, survival, and apoptosis; HIF1A can also promote tumor cell migration in different tumors [12]. Vascular endothelial growth factor (VEGF), accepted as the target gene of HIF1A, is one of the most potent angiogenic factors [13]. The HIF1A/VEGFA axis has been found to play a crucial role in angiogenesis [14]. Under hypoxia, human umbilical vascular endothelium cells (HUVECs) and human dermal microvascular endothelial cells (HDMECs) are regulated by the HIF1A/miR-210/miR-424/solube fms-related tyrosine kinase 1 (sFLT1) axis [15]. Additionally, HIF1A can regulate angiogenesis in trophoblastic cells through the Notch1/STAT3/ETBR pathway [16]. HIF1A can promote the paracrine function of ADSCs and the angiogenesis of HDMECs via targeting miR-20a expression. However, little is known about whether there is a regulatory relationship between HIF1A and LINC02913.

In the present study, the GSE12884 microarray dataset was first downloaded from Gene Expression Omnibus (GEO) database. Differentially expressed lncRNAs in ADSCs under normoxia and hypoxia in microarray dataset GSE12884 were identified. LINC02913 was found noticeably downregulated in ADSCs cultured under hypoxia. Restoring LINC02913 expression could inhibit the proliferation, adipogenic ability, endothelial differentiation ability, and tube formation ability of ADSCs, as well as the expression of endothelial cell markers. Furthermore, HIF1A targeted LINC02913 transcription to promote insulin-like growth factor 1 receptor (IGF1R) expression and the PI3K/AKT pathway activation, thereby facilitating the cell function of ADSCs under hypoxia. Finally, a skin wound healing nude mice model was established and the regulatory effects of LINC02913/IGF1R axis on hypoxia-ADSCs treated skin wound healing were verified.

## Materials and methods

### Bioinformatics analysis

The GSE12884 microarray dataset was downloaded from the GEO database (https://www.ncbi.nlm.nih.gov/gds). The microarray contained the expression profiling of ADSCs from 6 female individuals cultured under different oxygen tensions (normal, 15%, 10%, 5%, and 1%) for 14 days [17]. The potential lncRNA-interacted proteins were predicted through the online database RNACT (https://rnact.crg.eu/). Next, the predicted LINC02913 target genes were subjected to pathway enrichment analysis using Metascape (https://metascape.org/) (a web-based tool for gene functional annotation) [18], with Min Overlap = 3, *p*-Value Cutoff = 0.01, and Min Enrichment = 1.5.

### Cell culture, treatment, and induction for differentiation

Human ADSCs (HUXMD-01001, Cyagen Biotechnology Co., Ltd., Guangzhou, China) were cultured in OriCell human ADSCs complete culture medium (HUXMD-90011, Cygen) in an incubator (37 ℃, 5% CO_2_) (Thermo, Mass., USA).

ADSCs were cultured under hypoxia and normoxia. For hypoxia treatment, cells were cultured in a multi-gas incubator (InvivO2; Baker Ruskinn, Sanford, ME, USA) at 37 ℃ in an atmosphere containing 5% CO_2_ balanced with nitrogen to reach different oxygen extensions (15, 10, 5, or 1%) for 48 h. For normoxia treatment, cells were cultured in a standard incubator containing 5% CO_2_ and 20% O_2_.

Next, ADSCs were induced for adipogenic differentiation. In short, after confluence, ADSCs underwent 14-day induction in adipogenic differentiation medium (HUXMD-90031, Cyagen) as per the manufacturer’s instructions. Next, cells were fixed in phosphate buffer saline (PBS) containing 10% formaldehyde solution and then subjected to oil red O staining for the visualization of lipid droplets.

ADSCs were induced for endothelial differentiation as previously described [19]. Briefly, ADSCs were seeded onto 100-mm dishes or 6-well plates at 40–60% confluence in ADSCs complete medium. The next day, following discarding the culture media by aspiration, cells were washed with PBS and added with endothelial cell growth medium 2 (EGM2) plus 10 ng/mL fibroblast growth factor 2 (FGF2). The induction for endothelial differentiation proceeded for 7 days, with the medium refreshed every 3 days. Then, cells were harvested for further investigation.

### Cell transfection

The full length of LINC02913 and coding sequence (CDS) of HIF1A and IGF1R were amplified by polymerase chain reaction (PCR) and cloned into pcDNA3.1-flag empty vector. Primers used for plasmid construction are listed in Additional file 2: Table S1. si-HIF1A and its negative control (si-NC) were supplied by Guangzhou RiboBio Co., Ltd. (Guangzhou, Guangdong, China). When growing to 70–80% confluence in 6-well plates, cells were transfected using Lipofectamine 2000 reagent (Invitrogen, Carlsbad, CA, USA) as per the instructions. After transfection for 48 h, functional experiments were carried out and cell RNA and total protein were extracted. Cells were grouped according to the overexpression plasmid or siRNA they were transfected.

### Cell counting kit-8 (CCK-8) assay

Cells in each treatment group were resuspended in the complete culture medium of ADSCs for single-cell suspension preparation. Cells were seeded onto 96-well plates (5000 cells/well), with 100 μL culture medium per well. Cells with different treatments were cultured for 48 h for the subsequent cell viability detection. Briefly, cells in each well were added with 10 μL CCK-8 solution, followed by further incubation for 2 h. Finally, the optical density (OD) value at 450 nm wavelength was measured.

### Tube formation assay

ADSCs with different treatments were cultured in EGM2 with FGF2 for 6 days before the tube formation assay. In short, the 96-well plates were pre-coated with matrix gel (10 mg/mL, 0.05 mL/well). Cells were incubated at 37 ℃ for 30 min, waiting for coagulation. Approximately 1 × 10^4^ cells in 100 μL of EGM2 were then seeded into each well and incubated at 37 ℃. After 6 h, the development of capillary-like networks was examined by phase-contrast microscopy and photographed. The tube formation was analyzed using ImageJ.

### Quantitative real-time PCR (qRT-PCR)

ADSCs were harvested; cell total RNA was extracted using TRIzol (Invitrogen). Reverse transcription was performed using the reverse transcription kit (TaKaRa, Tokyo, Japan) following the instructions. Quantitative PCR for gene expression detection was performed on a LightCycler 480 system (Roche, Indianapolis, IN, USA) using the SYBR Green Mix (Roche Diagnostics) as per the corresponding instructions. The reaction conditions were: 95 ℃ for 10 s, and then 40 cycles of 95 ℃ for 5 s, 60 ℃ for 10 s, and 72 ℃ for 10 s, followed by a final extension at 72 ℃ for 5 min. Three replicates of quantitative PCR were performed for each sample. Glyceraldehyde-3-phosphate dehydrogenase (GAPDH) served as the internal reference. Data were analyzed using the 2^−ΔΔCt^ method; ΔΔ Ct = experimental group [Ct (target gene)—Ct (reference gene)]—control group [Ct (target gene)—Ct (reference gene)]. The primer sequences are shown in Additional file 2: Table S2.

### Western blot assay

Cells were lysed using the RIPA lysis buffer (Beyotime Biotechnology Co., Ltd., Shanghai, China) to obtain protein samples. The protein concentration was measured using the BCA kit (Beyotime). Next, protein samples with the corresponding volume were added and mixed with the loading buffer (Beyotime), which was then heated in a boiling water bath for 5 min for protein denaturation. After SDS-PAGE electrophoresis, the protein was transferred from the gel to membranes. After that, the membranes were washed for 1–2 min in the TBST solution and incubated (room temperature, 60 min) with the 5% non-fat milk TBST solution. Next, the membranes were incubated (4 ℃, overnight) on the shaking table with the primary antibodies [GAPDH (5174S, 1:1000, Cell Signaling Technology (CST), Boston, USA), cluster of differentiation (CD)31 (#3528, 1:1000, CST), CD133 (#64326, 1:1000, CST), VEGFA (#50661, 1:1000, CST), endothelial nitric oxide synthase (eNOS) (#32027, 1:1000, CST), HIF1A (# 36169, 1:1000, CST), p-PI3K (#17366, 1:1000, CST), PI3K (#4249, 1:1000, CST), p-AKT (#4060, 1:1000, CST), AKT (#4685, 1:1000, CST), TGF-β1 (#3711, 1:1000, CST), and α-Tubulin (#3873, 1:1000, CST). The next day, the membranes were washed 3 times, 10 min for each time. After that, the secondary antibody horseradish peroxidase-labeled goat anti-rabbit or anti-mouse IgG (1:5000, Proteintech, China) was added for incubation (room temperature, 1 h). After 3 washings, with 10 min each, the membranes were developed using the chemiluminescence imaging system (Tannon-5200, China).

### Immunofluorescence (IF) staining

Cells with different treatments were prepared into cell suspension. Next, cell slides at the density of 1 × 10^5^/mL were inoculated in the 24-well plates. After the cells were adhered to the wall for 24 h, IF staining was performed. In short, cells were fixed in 4% paraformaldehyde solution at room temperature for 30 min, washed 3 times in PBS, and then added with 0.1% TritonX-100 solution for 20 min at room temperature. Following 3 times of PBS rinsing, cells were treated with 10% goat serum for blocking (room temperature, 30 min). Next, goat serum was discarded and soaked up without rinsing. CD31 (#3528, 1:100, CST) diluted with 10% goat serum (ZSGB-Bio Co., Ltd, Beijing, China) was added for incubation (4 ℃, overnight) in a refrigerator away from light. After PBS rinsing 3 times, and then PBS absorption using filter paper, 1 mL fluorescent secondary antibody (ab150113 and ab150115, 1:500, Abcam, Mass., USA) diluted with 10% goat serum was added for incubation (room temperature, 1 h at dark). Next, cells were rinsed in PBS for 3 times, and then PBS was absorbed with filter paper. After that, cells were stained (room temperature, 1 min) with 1 mL 4′,6-diamidino-2-phenylindole (DAPI) staining solution (ab104139 Abcam) and rinsed with PBS for 3 times. After that, 1 mL of anti-fluorescence quenching sealing solution was added to the culture dish. The fluorescence microscope (200 ×) was used for observation; pictures were photographed.

### Chromatin immunoprecipitation (ChIP) assay

ChIP assay was performed using the ChIP kit (#56383, CST) following the instructions. Briefly, DNA-binding proteins were cross-linked to DNA with 1% formaldehyde, which was then terminated by the addition of glycine. Cells were lysed and chromatin was sheared by sonication. The input sample was prepared with 2% of the sheared chromatin. DNA-contained chromatin was subjected to immunoprecipitation with an anti-HIF1A antibody or IgG according to the manufacturer’s protocol. Protein G magnetic beads were added for precipitation. The precipitate was washed several times. The protein-DNA crosslinks were reversed, and DNA was purified. The obtained DNA was used as the template for real-time PCR amplification of ERE on the LINC02913 promoter. The primer sequence is shown in Additional file 2: Table S3.

### Dual-luciferase reporter gene assay

The binding sites between HIF1A and LINC02913 promoter were predicted through the online database JASPAR (https://jaspar.genereg.net/analysis). Based on the predicted results, the wild sequence and mutation sequence of the binding site (psicheck2-proLINC02913-WT, psicheck2-proLINC02913-MUT) were designed and synthesized respectively. The wild-type and mutant sequence of the binding sites were cloned into the luciferase reporter gene vector, respectively. Next, these constructed vectors were co-transfected with the control vector or HIF1A into HEK 293 T cells. Cells were added with 100 µL cell lysis buffer and placed on a shaking table at room temperature for 20 min for complete cell lysis. Next, 50 µL lysed cell suspension was added with 50 µL luciferase reaction solution (Promega, Madison WI, USA) for the determination of Firefly luciferase activity. Cells were added and mixed with 50 µL Stop&Glo reagent (Promega) for the determination of Renilla luciferase activity. Renilla luciferase activity was used as the internal reference. The relative luciferase activity was the ratio of Firefly luciferase activity to Renilla luciferase activity. Three replicates were set for the experiment. Primers used for plasmid construction are shown in Additional file 2: Table S4.

### Wound healing model

All the animal experiments procedures were approved by the Ethic Committee of the Second Xiangya Hospital. Forty-two nude mice (male, six-week-old) were assigned into seven groups (N = 6 per group): i. spontaneous healing group (model control group); ii. normoxia-ADSCs group (model mice were treated with normoxia-ADSCs); iii. hypoxia-ADSCs group (model mice were treated with hypoxia-ADSCs); iv. hypoxia-ADSCs+ vector group (model mice were treated with hypoxia-ADSCs which were transfected with empty vector); v. hypoxia-ADSCs+ LINC02913 OE group (model mice were treated with hypoxia-ADSCs which were transfected with LINC02913 overexpression vector); vi. hypoxia-ADSCs+ IGF1R OE group (model mice were treated with hypoxia-ADSCs which were transfected with IGF1R overexpression vector); vii. hypoxia-ADSCs+ LINC02913 OE+ IGF1R OE group (model mice were treated with hypoxia-ADSCs which were transfected with LINC02913 and IGF1R overexpression vectors). Based on previous research reports and make some modifications [20–22], forty-two nude mice (male, six-week-old) were anesthetized and surgery was performed under standard sterile conditions. One circular, full thickness 8 mm diameter wound was created on the back of each mouse and a ring of silicone was sutured in place to prevent skin retraction. During the follow-up, buprenorphine was administrated intra-peritoneally every day at a dose of 0.1 mg/kg/day to avoid suffering. A total of 15 × 10^5^ cells ADSCs in 100 μL of PBS were transplanted intradermally at four injection sites on the border between the wound and the normal skin. Mice were observed and digital images were taken; and then wound area was measured by tracing the wound margin and calculating the pixel area using image analysis software.

### Histological analysis

For hematoxylin and eosin (H&E) staining, the 4% paraformaldehyde-fixed, paraffin-embedded skin tissues were sectioned at a thickness of 4 μm. After being dewaxed in xylene and hydrated in gradient alcohol, tissue slices were stained using H&E staining kit (Servicebio, Wuhan, China) according to the manufacturer’s protocol. A microscope (Olympus, Tokyo, Japan) was used to visualize sections after blocking with neutral resin.

For Immunohistochemistry (IHC) staining, after antigen retrieval, skin tissue sections were incubated with 3% H_2_O_2_ for 15 min and further incubated with a blocking solution for 30 min, followed by 2-h incubation with CD31 (ab182981, 1:1000, Abcam) antibody, 30-min incubation at 37 ℃ with a secondary antibody horseradish peroxidase-labeled goat anti-rabbit IgG (1:5000, Proteintech, China). Next, sections were incubated with freshly prepared DAB reagent (Beyotime) for 10 min and then subjected to a counterstaining with hematoxylin (Beyotime). Finally, a light microscopy (Olympus) was employed to visualize the sections.

### Statistical analysis

The obtained experimental data were expressed as mean ± standard deviation (SD). One-way analysis of variance (ANOVA) followed Tukey test was performed using GraphPad Prism 8.0 software. A *p*-value of less than 0.05 represented a statistically significant difference.

## Results

### Differentially expressed lncRNAs in ADSCs under hypoxia were identified

**Fig. 1:**
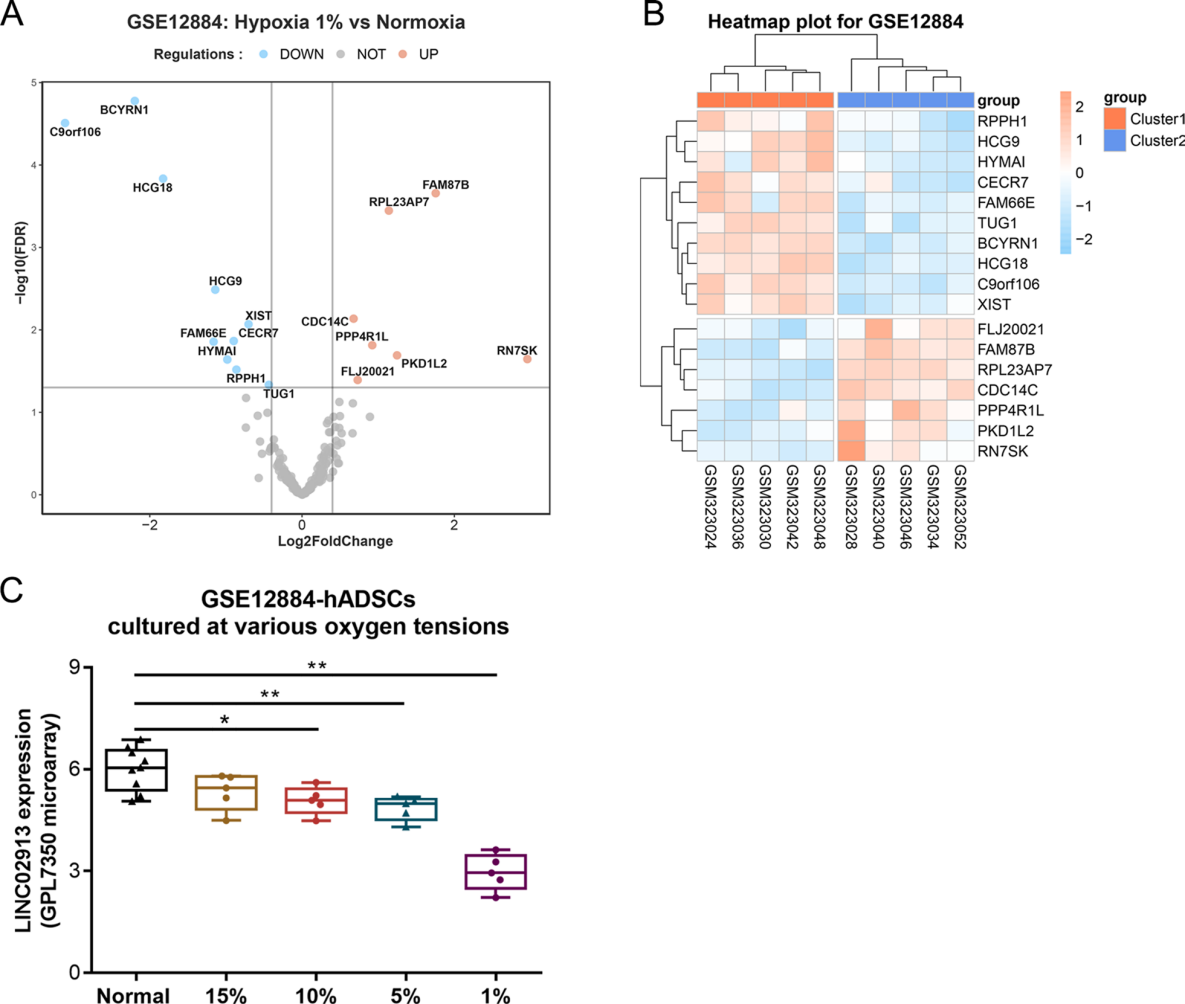
Differentially expressed lncRNAs in ADSCs under hypoxia were identified. Differentially expressed lncRNAs in microarray GSE12884 were displayed using volcanic map (**A**) and heatmap (**B**); **C** LINC02913 expression in ADSCs under different oxygen extensions in microarray GSE12884 was analyzed. *p < 0.05, **p < 0.01 compared to normal group

Differentially expressed lncRNAs in ADSCs incubated for 14 days under 1% O_2_ hypoxia or normoxia in microarray GSE12884 were screened. A total of 50 lncRNAs were identified, with the screening conditions of |logfc|> 0.4 and adjusted *p*.value < 0.05. According to the results, there were 7 upregulated lncRNAs and 10 downregulated lncRNAs (Table 1, Fig. 1A); C9orf106 (LINC02913) showed the most significant downregulation under 1% O_2_ hypoxia (logfc = − 3.11, *p*.value = 2.3e-07) (Fig. 1B). LINC02913 expression is positively correlated with the concentration of oxygen content; LINC02913 expression was decreased with the decrease of oxygen content, with LINC02913 expression at the lowest under 1% O_2_ hypoxia (Fig. 1C).

**Table 1:** Differentially expressed lncRNAs in ADSCs incubated for 14 days under 1% O2 hypoxia or normoxia in microarray GSE12884

	LogFc	AveExpr	t	p.Value	Adj.p.Val	B	Change
BCYRN1	− 2.19669	11.54786	− 10.544	7.41E-08	1.67E-05	8.571328	DOWN
LINC02913	− 3.11254	4.518581	− 9.58867	2.30E-07	3.10E-05	7.455231	DOWN
HCG18	− 1.8254	5.863946	− 7.7036	2.82E-06	1.46E-04	4.947379	DOWN
FAM87B	1.754801	4.863964	7.286903	5.19E-06	2.20E-04	4.33173	UP
RPL23AP7	1.139683	8.466983	6.775276	1.13E-05	3.58E-04	3.542972	UP
HCG9	− 1.14024	3.5626	− 4.93525	2.50E-04	3.26E-03	0.400354	DOWN
CDC14C	0.676608	5.838084	4.351308	7.36E-04	7.32E-03	− 0.68834	UP
XIST	− 0.70284	9.87884	− 4.23609	9.14E-04	8.54E-03	− 0.90705	DOWN
CECR7	− 0.89756	8.208767	− 3.90968	1.70E-03	1.37E-02	− 1.53167	DOWN
FAM66E	− 1.1615	3.470574	− 3.89683	1.75E-03	1.39E-02	− 1.55637	DOWN
PPP4R1L	0.922061	4.71182	3.826835	2.00E-03	1.54E-02	− 1.69108	UP
PKD1L2	1.247699	5.335711	3.63585	2.89E-03	2.04E-02	− 2.05931	UP
RN7SK	2.959101	4.319389	3.563655	3.32E-03	2.26E-02	− 2.19862	UP
HYMAI	− 0.98166	3.472993	− 3.55434	3.38E-03	2.29E-02	− 2.21659	DOWN
RPPH1	− 0.86227	4.344066	− 3.36431	4.90E-03	3.03E-02	− 2.58297	DOWN
FLJ20021	0.730147	5.39543	3.163578	7.24E-03	4.06E-02	− 2.96841	UP
TUG1	− 0.43652	11.91482	− 3.07372	8.63E-03	4.64E-02	− 3.14002	DOWN

### LINC02913 expression affected ADSC cell phenotype under hypoxia or normoxic

**Fig. 2:**
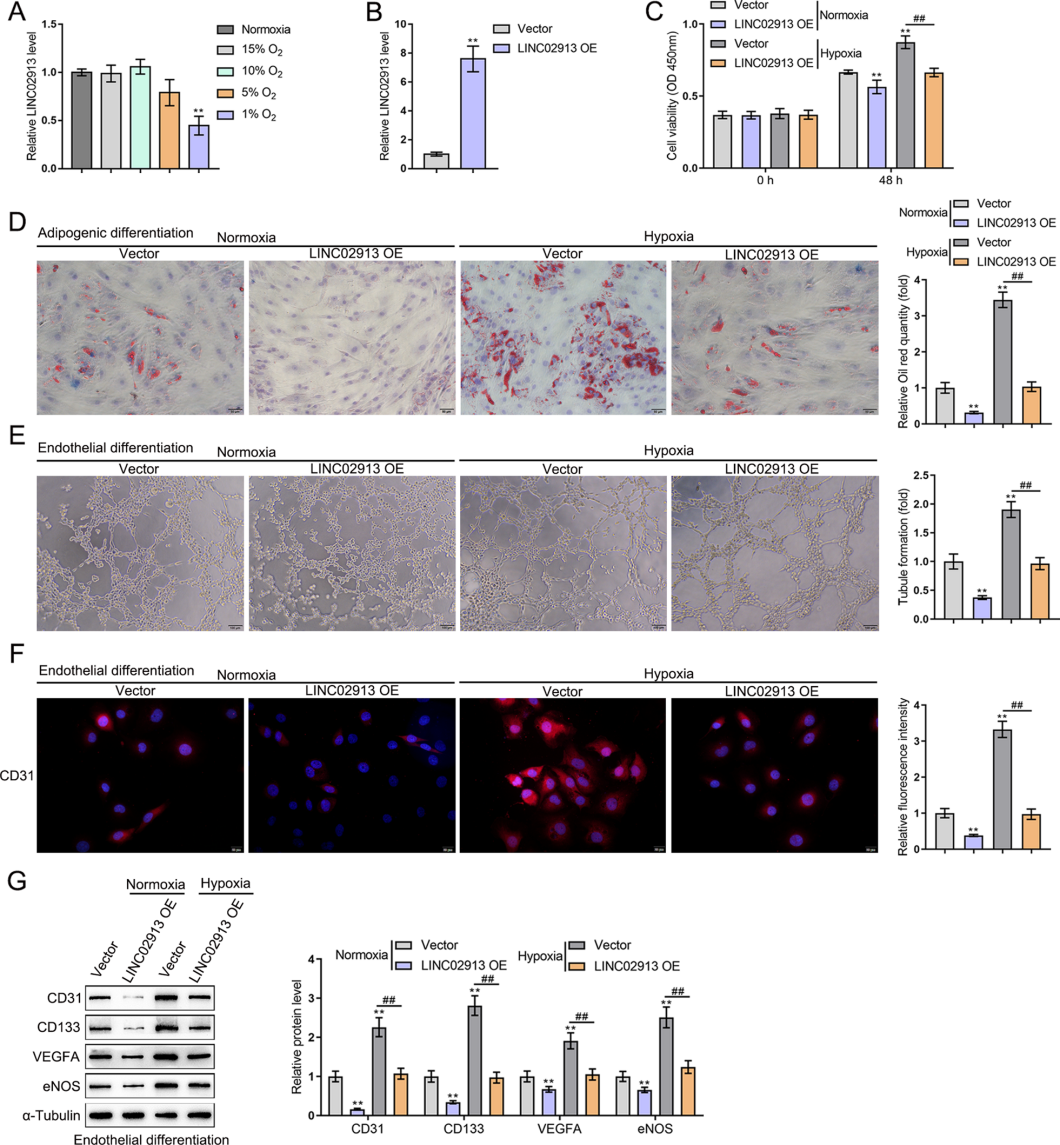
LINC02913 expression affected ADSC cell phenotype under hypoxia and normoxia. **A** LINC02913 expression in ADSCs cells treated with different oxygen extensions was detected using qRT-PCR; **B** the transfection efficiency of LINC02913 overexpression plasmid (LINC02913 OE) was verified using qRT-PCR; **C** the changes in cell viability after LINC02913 overexpression under hypoxia and normoxic conditions were detected using CCK-8 assay; **D** the changes in the adipogenic ability of cells after LINC02913 overexpression under hypoxia and normoxic conditions were detected using oil red O staining; **E** after the induction for endothelial differentiation of ADSCs, cell tube formation after LINC02913 overexpression under hypoxia and normoxic conditions were detected using the tube formation assay; **F** the expression of endothelial cell marker CD31 was detected using IF staining after LINC02913 overexpression under hypoxia and normoxic conditions; **G** the level of endothelial cell markers (CD133, CD31, VEGFA, and eNOS) after LINC02913 overexpression under hypoxia and normoxic conditions was detected using western blot. **p < 0.01 compared to Nypoxia + vector group; ##*p* < 0.01, compared with Hypoxia+vector group

ADSCs were cultured under different oxygen concentrations for 48 h and then examined for LINC02913 expression using qRT-PCR. Consistent with the results of microarray GSE12884, LINC02913 expression was remarkably decreased under 1% O_2_ hypoxia compared with that under normoxia (*p* < 0.01) (Fig. 2A). Subsequently, the LINC02913 overexpression plasmid (LINC02913 OE) was constructed, and then transfected into ADSCs, with the transfection efficiency verified using qRT-PCR (*p* < 0.01) (Fig. 2B). Next, cell viability was detected using CCK-8 assay under hypoxia or normoxic conditions. It was found that hypoxia stimulation notably promoted cell viability compared to normoxic and LINC02913 OE noticeably reduced cell viability in both hypoxia and normoxic conditions (*p* < 0.01) (Fig. 2C). Cell adipogenic ability was detected using oil red O staining under hypoxia or normoxic conditions. As shown by the results, hypoxia notably promoted adipogenic ability compared to normoxic and LINC02913 overexpression notably reduced adipogenic ability in both hypoxia and normoxic conditions (*p* < 0.01) (Fig. 2D). Next, ADSCs were transfected with LINC02913 OE and induced for endothelial differentiation for 2 weeks under hypoxia or normoxic and cell tube formation was detected. It was found that hypoxia notably promoted tube formation ability compared to normoxic and LINC02913 overexpression could remarkably reduce the number of cell tubes in both hypoxia and normoxic conditions (*p* < 0.01) (Fig. 2E). Moreover, after endothelial differentiation induction, the expression of CD31, a marker of endothelial cells, was detected using IF staining. According to the IF staining results, hypoxia notably promoted CD31 expression when compared with normoxic; CD31 expression was dramatically reduced in LINC02913 overexpression in both hypoxia and normoxic conditions (*p* < 0.01) (Fig. 2F). Then, the level of CD133, CD31, VEGFA, and eNOS (endothelial cell differentiation markers) was detected using western blot. The results showed that hypoxia notably promoted CD133, CD31, VEGFA, and eNOS protein levels when compared with normoxic; CD133, CD31, VEGFA, and eNOS protein levels were notably reduced in LINC02913 overexpression in both hypoxia and normoxic conditions (all *p* < 0.01) (Fig. 2G). The above results indicated that LINC02913 expression was decreased in ADSCs under hypoxia and normoxic conditions. LINC02913 overexpression could inhibit the proliferation viability, adipogenic ability, and tube formation of ADSCs, as well as the expression of endothelial cell markers in ADSCs.

### HIF1A bound to LINC02913 promoter region to inhibit its expression

**Fig. 3:**
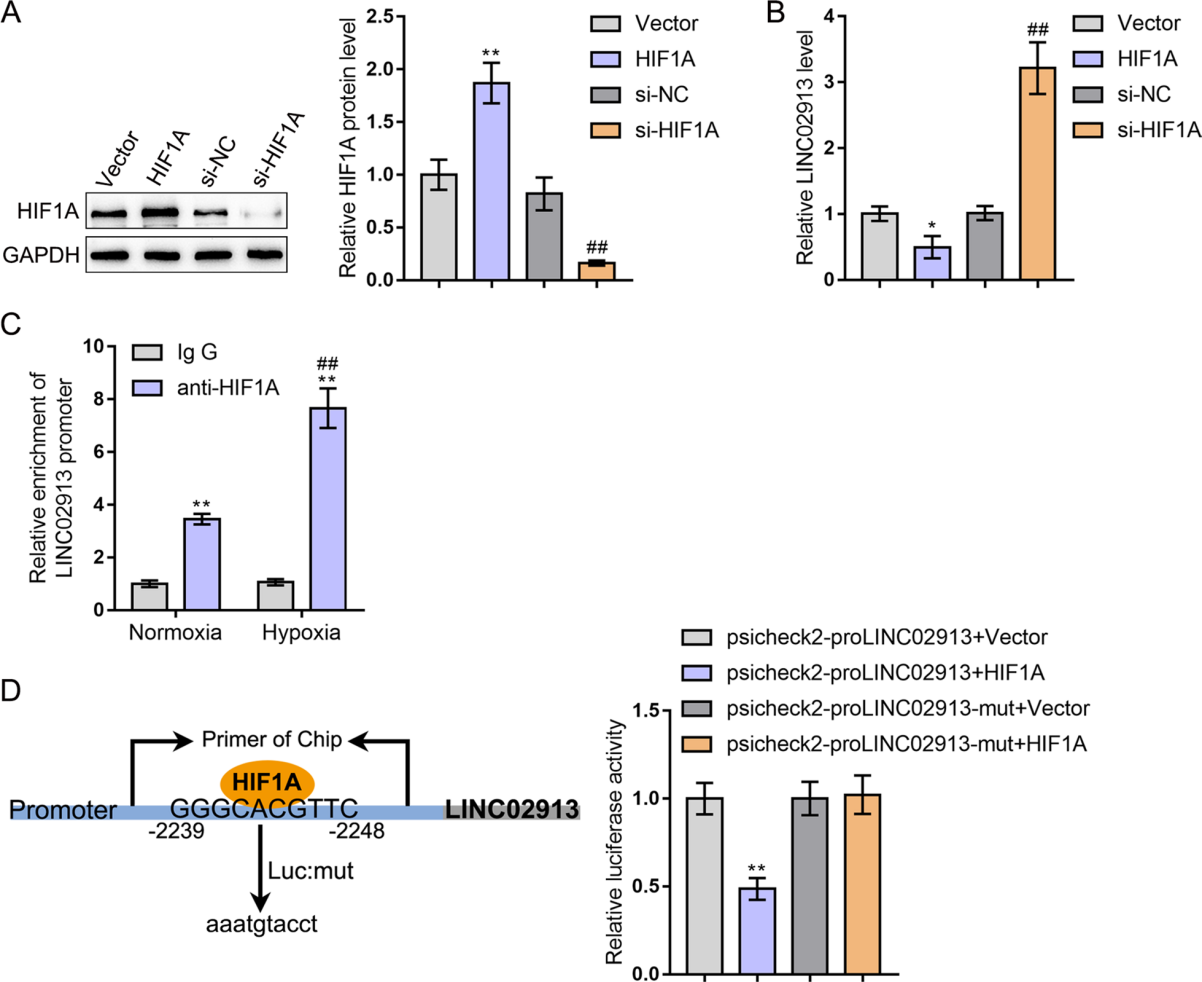
HIF1A bound to LINC02913 promoter region to inhibit its expression. **A** The transfection efficiency of HIF1A overexpression plasmid (HIF1A) and siRNA (si-HIF1A) was detected using western blot; **B** LINC02913 expression was detected after HIF1A overexpression and knockdown using qRT-PCR; **C** the binding of HIF1A with LINC02913 promoter region was verified using ChIP assay; **D** the binding between HIF1A and LINC02913 promoter region was verified using the dual-luciferase reporter gene assay; **p < 0.01, compared with the vector group; ##*p* < 0.01, compared with the si-NC group

Firstly, the binding sites between the promoter region of LINC02913 and HIF1A were predicted through the JASPAR website (Table 2). The transfection efficiency of HIF1A overexpression plasmid (HIF1A) and siRNA (si-HIF1A) was detected using western blot (Fig. 3A). Next, LINC02913 expression was detected after HIF1A overexpression and knockdown using qRT-PCR (Fig. 3B). As shown by western blot and qRT-PCR results, HIF1A overexpression could increase HIF1A protein level and decrease LINC02913 expression compared with the vector group (both *p* < 0.05); HIF1A knockdown effectively decreased HIF1A protein level and increased LINC02913 expression compared with the si-NC group (both *p* < 0.01) (Fig. 3A, B). The binding of HIF1A with the LINC02913 promoter region was verified using a ChIP assay. According to the results, the abundance of LINC02913 promoter pulled down by anti-HIF1A was dramatically increased under both normoxia and hypoxia compared with that pulled down by a control IgG antibody (both *p* < 0.01) (Fig. 3C). Furthermore, the binding between HIF1A and LINC02913 promoter region was verified using the dual-luciferase reporter gene assay. It was observed that the luciferase activity of the LINC02913 promoter region was remarkably reduced after HIF1A overexpression (*p* < 0.01); however, no significant difference in the fluorescence activity of the mutant LINC02913 promoter region after HIF1A overexpression was observed (Fig. 3D). Taken together, HIF1A could directly bind to LINC02913 promoter region to inhibit LINC02913 transcription.

**Table 2:** Prediction of the binding sites between the LINC02913 promoter region and HIF1A through JASPAR

Matrix ID	Name	Score	Relative score	Sequence ID	Start	End	Strand	Predicted sequence
MA1106.1	MA1106.1.HIF1A	11.44319	0.961561	3	2239	2248	−	GAACGTGCCC
MA0259.1	MA0259.1.ARNT::HIF1A	9.656816	0.953992	3	2241	2248	−	GAACGTGC
MA0259.1	MA0259.1.ARNT::HIF1A	8.171119	0.90967	3	2103	2110	+	GTGCGTGG

### LINC02913 expression was negatively correlated with IGF1R expression

**Fig. 4:**
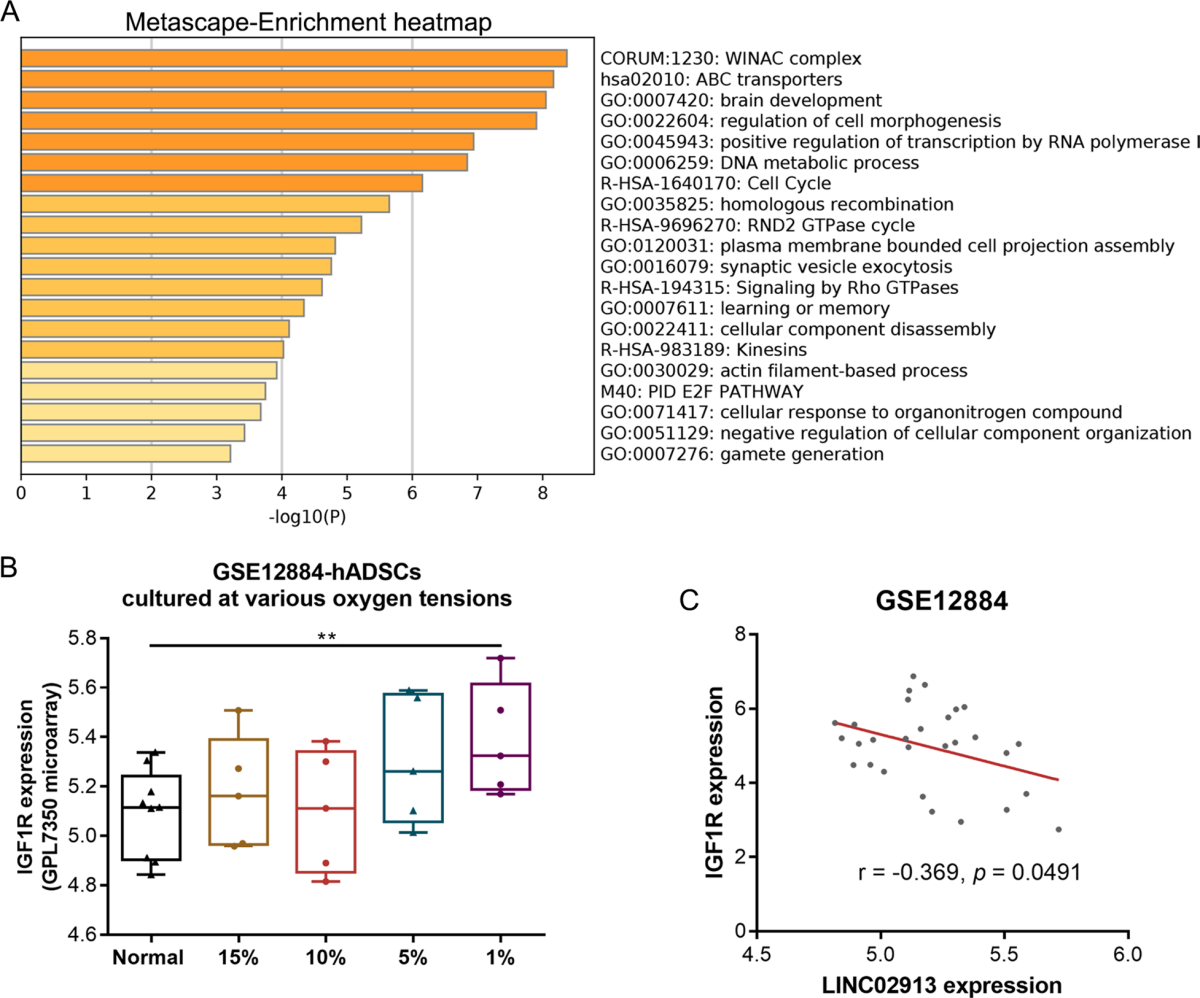
LINC02913 expression was negatively correlated with IGF1R expression. **A** A total of 117 target proteins binding to LINC02913 were predicted through RNAact; these target proteins were subjected to pathway enrichment analysis using the Metascape database; **B** IGF1R expression in ADSCs under different oxygen concentrations in GSE12884 microarray was displayed; **C** the correlation between IGF1R expression and LINC02913 expression in GSE12884 microarray was analyzed. **p < 0.01, compared with the Normal group

A total of 117 target proteins binding to LINC02913 were identified through prediction (prediction score > 20) by RNAact (https://rnact.crg.eu/) (Additional file 2: Table S5). Next, these target proteins were subjected to pathway enrichment analysis using the Metascape database. As indicated by the results, these 117 target proteins of LINC02913 were enriched in the pathways of morphogenesis, cell cycle, and DNA replication. Among these target proteins, there were 17 factors (CUX1, CUL7, CUX2, RAPGEF2, BCL11A, ARHGAP35, ARAP1, IGF1R, TNIK, GRIN2B, SYNJ1, ABCC8, TTBK1, WDR62, FGD5, UCA13A, and DNMBP) related to cell morphogenesis and tissue development (Fig. 4A). Moreover, IGF1R and CUL7 were identified to be differentially upregulated in the microarray GSE12884. On the other hand, a previous study has pointed out that IGF1R, as the ligand of growth factor IGF1, could affect the PI3K/AKT pathway in cells to participate in proliferation and angiogenesis [23]. Therefore, IGF1R was selected as the subsequent research object. IGF1R expression in ADSCs under different oxygen concentrations in the GSE12884 microarray was displayed. It was found that IGF1R expression was remarkably elevated in ADSCs under 1% O_2_ hypoxia (*p* < 0.01) (Fig. 4B). Moreover, IGF1R expression was found negatively correlated with LINC02913 expression in the GSE12884 microarray (Fig. 4C).

### LINC02913/IGF1R axis affected the phenotype of ADSCs under hypoxia or normoxic conditions

**Fig. 5:**
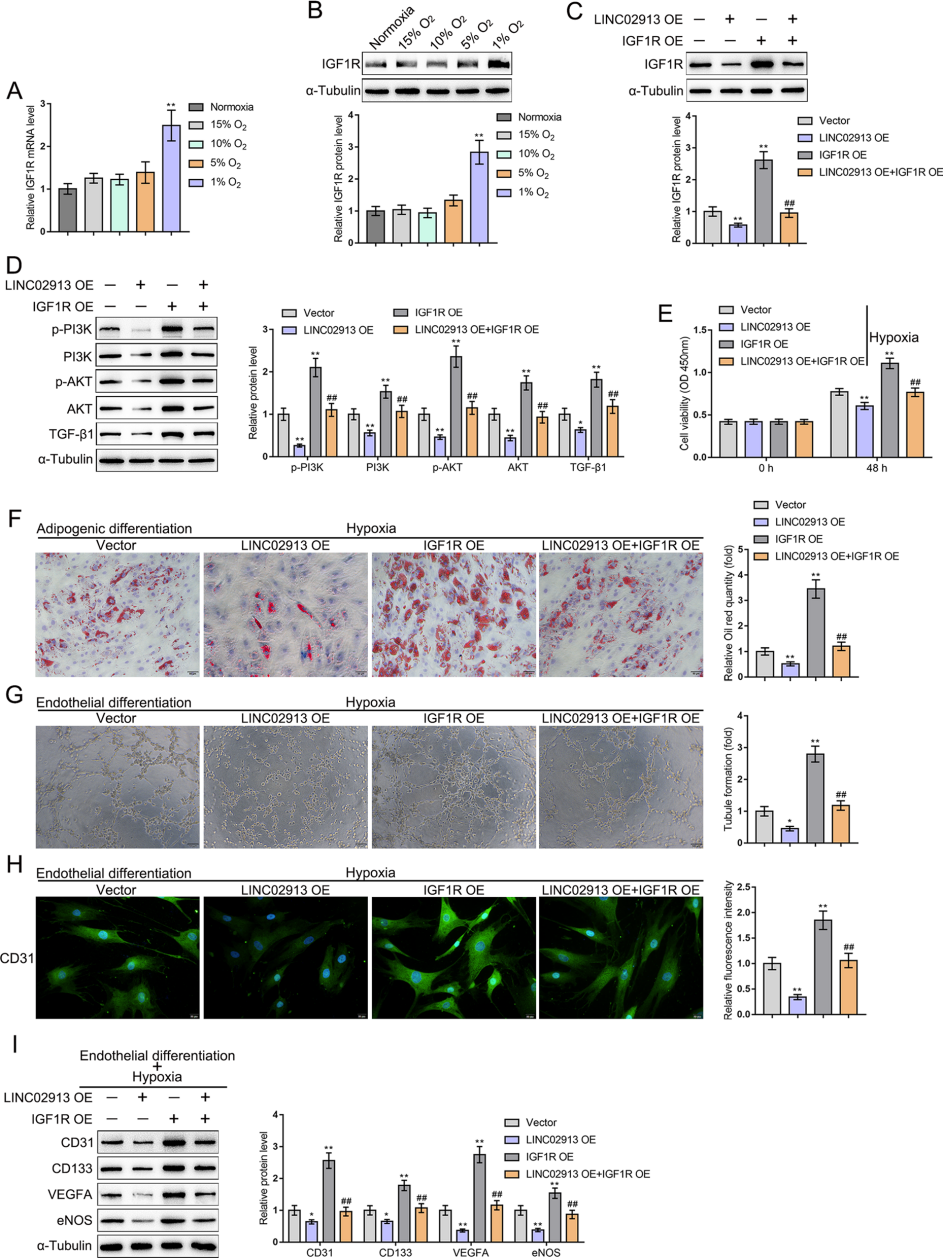
The LINC02913/IGF1R axis affected the phenotype of ADSCs under hypoxia via regulating the PI3K/AKT pathway. **A** IGF1R mRNA expression in ADSCs cultured under different oxygen concentrations was detected using qRT-PCR; **B** IGF1R protein level in ADSCs cultured under different oxygen concentrations was detected using western blot; **C** the effect of the LINC02913/IGF1R axis on IGF1R expression under hypoxia was detected through a series of rescue experiments using western blot; **D** the effect of the LINC02913/IGF1R axis on the PI3K/AKT pathway-related protein was detected using western blot; **E** the effect of the LINC02913/IGF1R axis on cell viability under hypoxia was detected using CCK-8 assay; **F** cell adipogenic ability under hypoxia was detected using oil red O staining; **G** after the endothelial differentiation of ADSCs, the effect of the LINC02913/IGF1R axis on cell tube formation under hypoxia was detected; **H** the effect of the LINC02913/IGF1R axis on the expression of CD31 (endothelial cell marker) was detected using IF staining; **I** the effect of the LINC02913/IGF1R axis on the level of endothelial cell markers CD133, CD31, VEGFA, and eNOS were detected using western blot. *p < 0.05, **p < 0.01, compared with the vector group; ##*p* < 0.01, compared with the LINC02913 OE group

Subsequently, IGF1R expression in ADSCs cultured under different oxygen concentrations was detected using qRT-PCR and western blot. Consistent with the microarray results, IGF1R mRNA expression and protein level were noticeably elevated in ADSCs under 1% O_2_ hypoxia compared with those in ADSCs under normoxia (both *p* < 0.01) (Fig. 5A, B). Next, the IGF1R overexpression plasmid (IGF1R OE) was constructed. The effect of the LINC02913/IGF1R axis on IGF1R expression under hypoxia was detected through a series of rescue experiments. According to western blot results, compared with the vector group, the overexpression of LINC02913 and IGF1R noticeably reduced and elevated IGF1R protein level, respectively; IGF1R overexpression could partially reverse LINC02913 overexpression-induced decrease of IGF1R protein level (all *p* < 0.01) (Fig. 5C). The effect of the LINC02913/IGF1R axis on the PI3K/AKT pathway-related proteins was detected using western blot. It was observed that compared with the vector group, the overexpression of LINC02913 and IGF1R could dramatically downregulate and upregulate the protein level of p-PI3K, PI3K, p-AKT, AKT, and TGF-β1, respectively; IGF1R overexpression could partially restore LINC02913 overexpression-caused decrease of p-PI3K, PI3K, p-AKT, AKT, and TGF-β1 protein levels (Fig. 5D). Cell viability was detected using CCK-8 assay under hypoxia condition. As shown by the results, IGF1R overexpression partially reversed the decrease of cell viability caused by LINC02913 overexpression (all *p* < 0.01) (Fig. 5E). ADSCs were transfected with LINC02913 OE and induced for adipogenic differentiation under hypoxia. Cell adipogenic ability was detected using oil red O staining. The results revealed that IGF1R overexpression could partially reverse the reduced cell adipogenic ability caused by LINC02913 overexpression (all *p* < 0.01) (Fig. 5F). ADSCs were transfected with LINC02913 OE and induced for endothelial differentiation for 2 weeks under hypoxia; then, cell tube formation was detected. As shown by the results, LINC02913 overexpression-caused reduction of cell tube formation could be partially reversed by IGF1R overexpression under hypoxic conditions (*p* < 0.01) (Fig. 5G). After LINC02913 overexpression and endothelial differentiation induction, the expression of CD31 (endothelial cell marker) was detected using IF staining. IGF1R overexpression could partially reverse the LINC02913 overexpression-induced decrease of CD31 expression (all *p* < 0.01) (Fig. 5H). LINC02913 was overexpressed under hypoxic hypoxia; the protein level of angiogenesis and endothelial cell markers CD133, CD31, VEGFA, and eNOS (representing the endothelial cell differentiation potential of ADSCs) was detected using western blot. According to western blot results, LINC02913 overexpression-caused decrease of CD133, CD31, VEGFA, and eNOS protein levels could be partially reversed by IGF1R overexpression (all *p* < 0.01) (Fig. 5I).

Moreover, the effects of LINC02913/IGF1R axis on the phenotype of ADSCs under normoxic condition were investigated (Additional file 1: Fig. S1). Under normoxic condition, LINC02913 overexpression notably inhibited, while IGF1R overexpression markedly promoted cell viability; IGF1R overexpression partially reversed the decrease of cell viability caused by LINC02913 overexpression (all *p* < 0.01) (Additional file 1: Fig. S1A). The oil red O staining results indicated that IGF1R overexpression could partially reverse the reduced cell adipogenic ability caused by LINC02913 overexpression under normoxic condition (all *p* < 0.01) (Additional file 1: Fig. S1B). Cell tube formation results showed LINC02913 overexpression-caused reduction of cell tube formation could be partially reversed by IGF1R overexpression under normoxic condition (*p* < 0.01) (Additional file 1: Fig. S1C). IF staining results displayed IGF1R overexpression could partially reverse the LINC02913 overexpression-induced decrease of CD31 expression under normoxic condition (all *p* < 0.01) (Additional file 1: Fig. S1D). Under normoxic condition, LINC02913 overexpression-caused decrease of CD133, CD31, VEGFA, and eNOS protein levels could be partially reversed by IGF1R overexpression (all *p* < 0.05) (Additional file 1: Fig. S1E). From all the above findings, IGF1R expression was increased in ADSCs under hypoxia. IGF1R overexpression could partially restore LINC02913 overexpression-inhibited proliferation, adipogenic ability, endothelial differentiation ability, and tube formation ability of ADSCs under hypoxia or normoxic conditions. The LINC02913/IGF1R axis exerted effects on the phenotype of ADSCs under hypoxia via regulating the PI3K/AKT pathway.

### Effects of hypoxic or normoxic-stimulated ADSCs on wound healing in nude mice

**Fig. 6:**
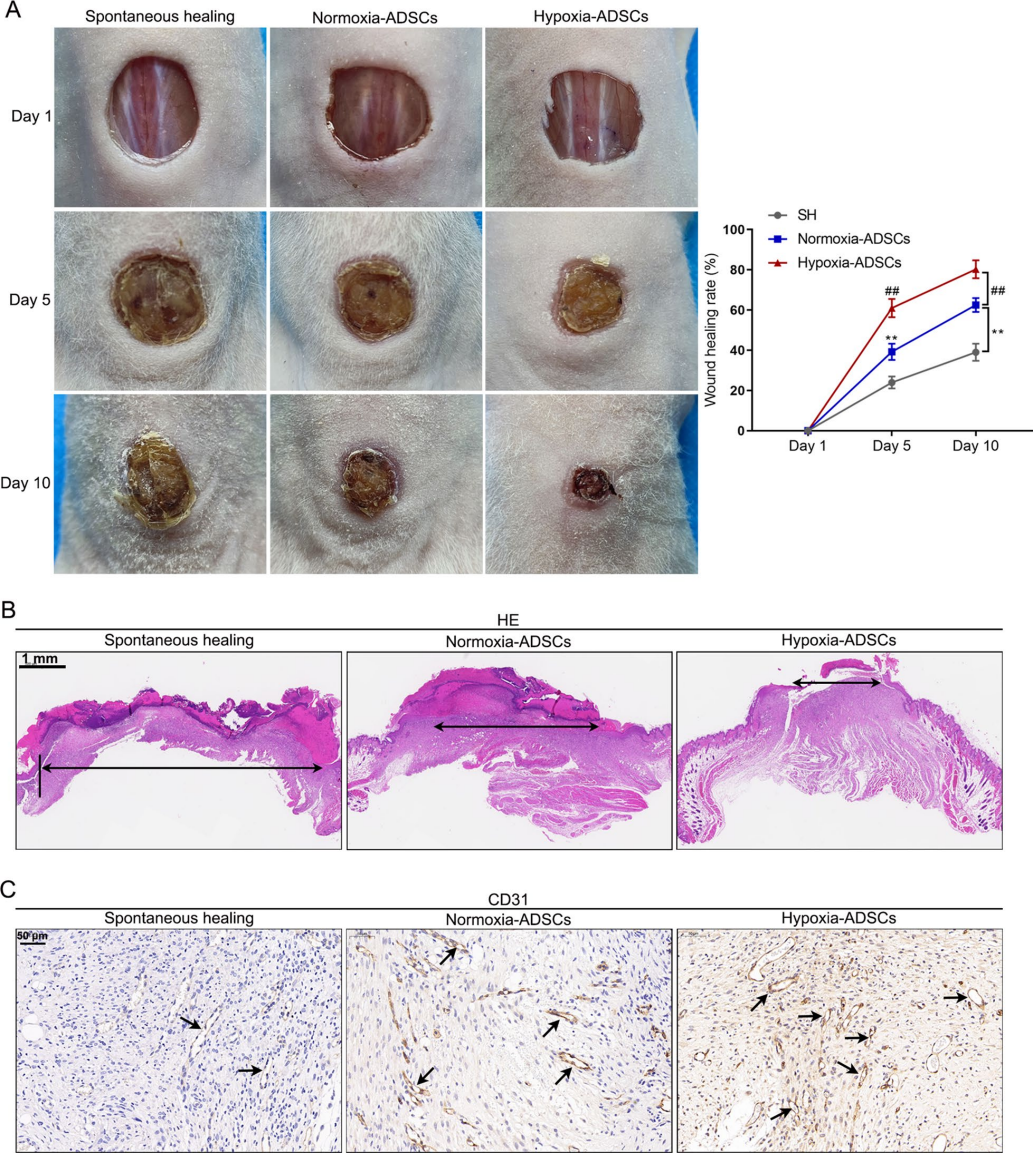
Effects of hypoxic or normoxic-stimulated ADSCs on wound healing in nude mice. The skin wound healing nude mice model was prepared and divided into 3 groups (N = 6 per group): spontaneous healing group (model control group); normoxia-ADSCs group (model mice were treated with normoxia-ADSCs); hypoxia-ADSCs group (model mice were treated with hypoxia-ADSCs). **A** skin wounds on days 1, 5, and 10 after wound puncture were exhibited, and then the percentage of wound area was calculated. **B** H&E staining was used to detect the pathological damage in wounded skin. **C** IHC staining was applied to detect CD31 expression in wounded skin. **p < 0.01, compared with spontaneous healing group; ##*p* < 0.01, compared with normoxia-ADSCs group

In vivo experiments utilizing nude mouse cutaneous wound healing model showed that application of ADSCs can significantly improve wound healing efficacy and the hypoxia-ADSCs group further accelerated wound closure (all *p* < 0.01) (Fig. 6A). Skin biopsy specimen from the wound (on day 10) was stained with H&E and CD31 antibody (Fig. 6B, C). In the model control group, H&E staining revealed a thin epidermis and inflammatory signs in the underlying dermis; hypoxia-ADSCs treated mice exhibited a thicker epidermis and normal underlying dermis (Fig. 6B). Furthermore, the number of CD31-positive cells in the hypoxia-ADSCs group was higher than that in the model control or normoxia-ADSCs group. These results suggest that hypoxia-ADSCs significantly promoted vascularization in the wounded skin.

### Effects of LINC02913/IGF1R axis on skin wound healing in ADSCs under hypoxia

**Fig. 7:**
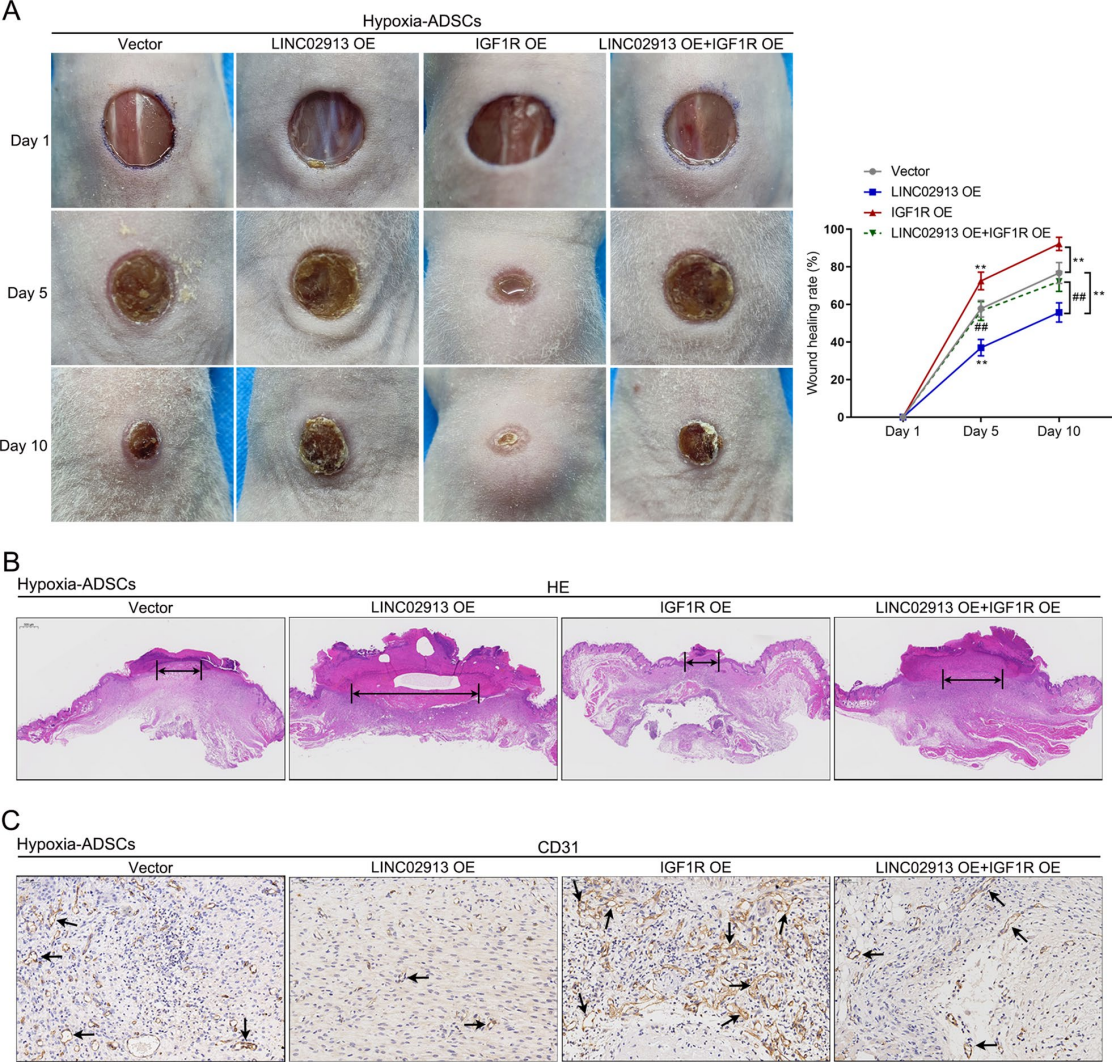
Effects of LINC02913/IGF1R axis on hypoxia-ADSCs treated skin wound healing. The skin wound healing nude mice model was established and divided into 4 groups (N = 6 per group): hypoxia-ADSCs+ vector group (model mice were treated with hypoxia-ADSCs which were transfected with empty vector); hypoxia-ADSCs+ LINC02913 OE group (model mice were treated with hypoxia-ADSCs which were transfected with LINC02913 overexpression vector); hypoxia-ADSCs+ IGF1R OE group (model mice were treated with hypoxia-ADSCs which were transfected with IGF1R overexpression vector); hypoxia-ADSCs+ LINC02913 OE+IGF1R OE group (model mice were treated with hypoxia-ADSCs which were transfected with LINC02913 and IGF1R overexpression vectors). **A** skin wounds on days 1, 5, and 10 after wound puncture were exhibited, and then the percentage of wound area was calculated. **B** H&E staining was used to detect the pathological damage in wounded skin. **C** IHC staining was applied to detect CD31 expression in wounded skin. **p < 0.01, compared with vector group; ##*p* < 0.01, compared with LINC02913 overexpression group

Finally, in vivo effects of LINC02913/IGF1R axis on skin wound healing in ADSCs under hypoxia were explored. The results showed that LINC02913 overexpression inhibited, while IGF1R overexpression accelerated wound closure. IGF1R overexpression partially offset the effect of LINC02913 overexpression on skin wound healing (all *p* < 0.01) (Fig. 7A). H&E results indicated that LINC02913 overexpression aggravated the skin pathological injury, while IGF1R overexpression enhanced re-epithelialization and granulation (Fig. 7B). Moreover, IGF1R overexpression suppressed CD31 expression, while IGF1R overexpression promoted CD31 expression in the skin tissues (Fig. 7C). These outcomes revealed that the regulatory effect of LINC02913/IGF1R axis on hypoxia-ADSCs treated skin wound healing.

## Discussion

ADSCs are endowed with strong differentiation potential and angiogenesis-promoting ability, which have been proposed as important seed cells for promoting tissue repair in stem cell therapy in regenerative medicine [24]. ADSCs can promote angiogenesis through the direct differentiation into endothelial cells and smooth muscle cells, paracrine secretion of various cytokines, growth factors, and chemokines, and release of exosomes/vesicles, showing a wide application prospect in treating ischemic injury and tissue repair. However, the survival and migration ability of stem cells in ischemic tissues are easily affected by local microenvironment factors, which causes the unstable therapeutic effects of stem cells.

Accumulating studies have demonstrated that lncRNAs can intrinsically regulate the biological functions of ADSCs. For example, lncRNA-Adi is highly expressed in ADSCs that are differentiating into adipocytes in obesity and diabetes; lncRNA-Adi knockdown impairs the adipogenic differentiation ability of ADSCs [25]. Restoring lncRNA-NEF (neighboring enhancer of FOXA2) expression can inhibit adipogenesis and promote osteogenesis in ADSCs via regulating the miR-155/PTEN axis in osteoporosis [26]. In the present study, LINC02913 expression was found downregulated in ADSCs under hypoxia; restoring LINC02913 expression could inhibit the adipogenic and tube formation ability of hypoxic-ADSCs in vitro and restrain the effect of hypoxic-ADSCs on skin wound healing in vivo.

HIF1A is accepted as an important hypoxia regulatory protein in the response to hypoxic stress. HIF1A can regulate the expression of various genes, including genes that code for angiogenic cytokines [such as VEGF, platelet-derived growth factor (PDGF), and angiopoietin-1 (Ang-1)] [27]. HIF1A expression can also affect the stemness, differentiation, and proliferation of MSCs via mediating multiple pathways (such as TGF-β, PI3k/Akt, Wnt, and Jagged/Notch) [28]. Moreover, HIF1A is downregulated in the skin of diabetes patients; HIF1A overexpression in ADSCs-derived extracellular vehicles can promote the healing rate and treatment of diabetes patients via activating the PI3K/AKT pathway [29]. In this study, after exploring the LINC02913 effect on the biological functions of ADSCs in vivo and in vitro, HIF1A was found to directly bind to the LINC02913 promoter region to inhibit LINC02913 transcription and activate the PI3K/AKT pathway, thereby promoting angiogenesis under hypoxia.

IGF1R is a tyrosine kinase cell surface receptor; the binding between IGF1R and its homologous ligand IGF1 or IGF2 contributes to activating two main downstream pathways (PI3K/Akt and RAS/MAPK pathways), thereby promoting cell proliferation, differentiation, migration, and survival, and inhibiting apoptosis [30]. Nevertheless, the role of IGF1R in ADSCs remains to be elucidated. In this study, IGF1R expression was for the first time found to be upregulated in ADSCs under hypoxic conditions; IGF1R could promote the biological function of ADSCs in vivo and in vitro. Regarding the mechanism, lncRNA significantly regulates gene expression both in the nucleus and cytoplasm. lncRNA could influence protein stability and inhibit expression at the post-transcriptional level [31, 32]. In our study, IGF1R upregulation in ADSCs under hypoxia was correlated with its upstream LINC02913 downregulation. Besides, IGF1R could affect the PI3K/AKT pathway in cells to participate in proliferation and angiogenesis [23]. Hence, the HIF1A/LINC02913/IGF1R axis promoted the adipogenesis and tube formation of ADSCs via activating the PI3K/AKT pathway.

All in all, the role of LINC02913 and IGF1R and the associated mechanism in ADSCs under hypoxia was explored for the first time. Increasing HIF1A expression may be helpful to improve the survival rate and proliferation and differentiation ability of ADSCs in ischemic tissues, thereby improving the therapeutic effect of stem cells.

### Supplementary Information

**Additional file 1: Figure S1. **The LINC02913/IGF1R axis affected the phenotype of ADSCs under normoxic condition. **A** the effect of the LINC02913/IGF1R axis on cell viability under normoxic was detected using CCK-8 assay; **B** cell adipogenic ability under normoxic was detected using oil red O staining; **C** after the endothelial differentiation of ADSCs, the effect of the LINC02913/IGF1R axis on cell tube formation under normoxic was detected; **D** the effect of the LINC02913/IGF1R axis on the expression of CD31 (endothelial cell marker) under normoxia was detected using IF staining; **E** the effect of the LINC02913/IGF1R axis on the level of endothelial cell markers CD133, CD31, VEGFA, and eNOS under normoxic were detected using western blot. *p < 0.05, **p < 0.01, compared with the vector group; #*p* < 0.05, ##*p* < 0.01, compared with the LINC02913 OE group.

**Additional file 2. **Supplementary Table S1–5.

## Data Availability

All data and materials are available.
