# Supplementary figures and images for: The HIFIA/LINC02913/IGF1R axis promotes the cell function of adipose-derived mesenchymal stem cells under hypoxia via activating the PI3K/AKT pathway
Source: J Transl Med. 2023 Oct 17;21:732. doi: 10.1186/s12967-023-04581-x (PMC10583486; doi:10.1186/s12967-023-04581-x)

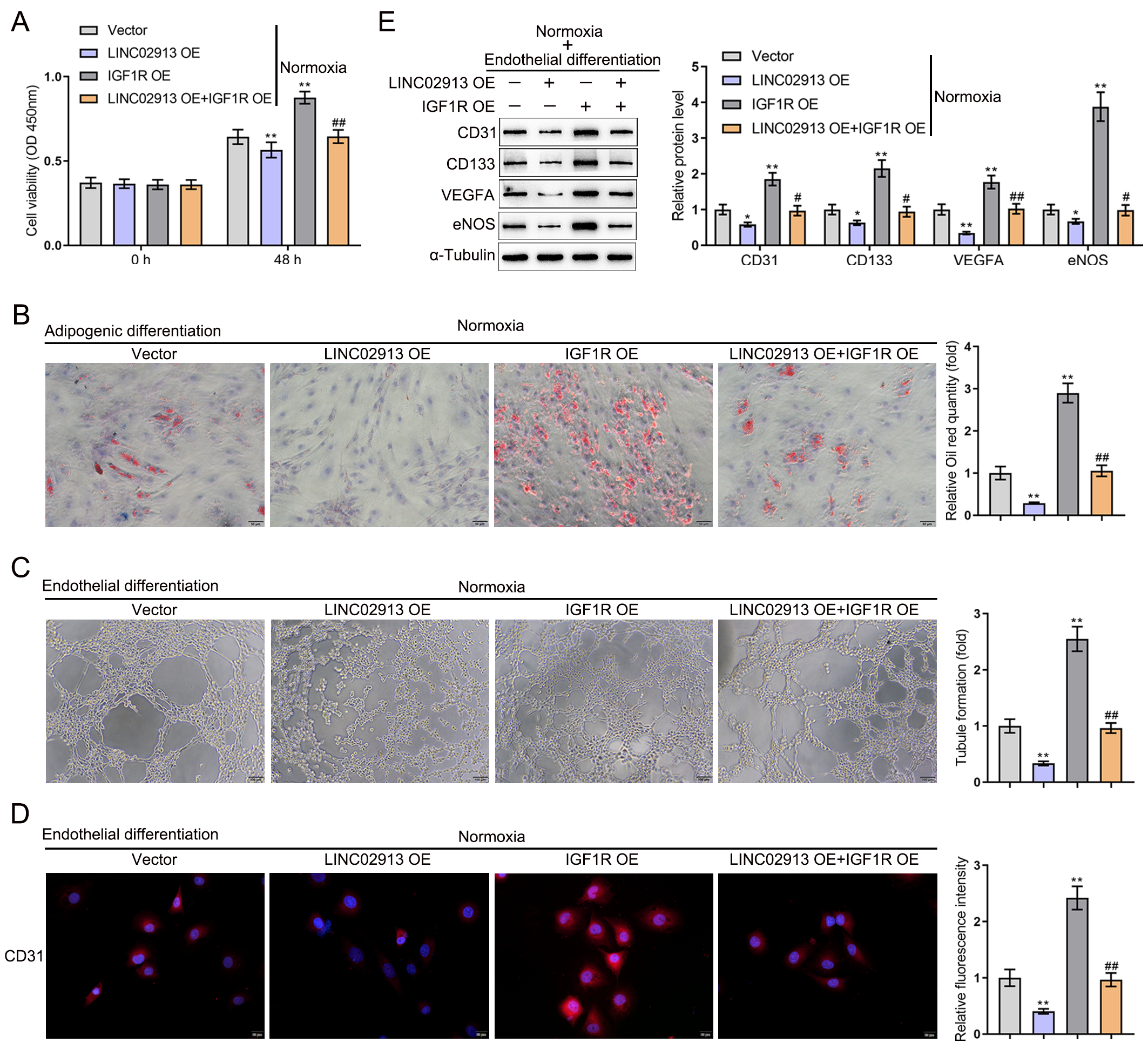

Supplement: Supplementary file 1 — Additional file 1: Figure S1. The LINC02913/IGF1R axis affected the phenotype of ADSCs under normoxic condition. A the effect of the LINC02913/IGF1R axis on cell viability under normoxic was detected using CCK-8 assay; B cell adipogenic ability under normoxic was detected using oil red O staining; C after the endothelial differentiation of ADSCs, the effect of the LINC02913/IGF1R axis on cell tube formation under normoxic was detected; D the effect of the LINC02913/IGF1R axis on the expression of CD31 (endothelial cell marker) under normoxia was detected using IF staining; E the effect of the LINC02913/IGF1R axis on the level of endothelial cell markers CD133, CD31, VEGFA, and eNOS under normoxic were detected using western blot. *p < 0.05, **p < 0.01, compared with the vector group; #p < 0.05, ##p < 0.01, compared with the LINC02913 OE group. [file 12967_2023_4581_MOESM1_ESM.tif]
