# Supplementary material for: The HIFIA/LINC02913/IGF1R axis promotes the cell function of adipose-derived mesenchymal stem cells under hypoxia via activating the PI3K/AKT pathway
Source: J Transl Med. 2023 Oct 17;21:732. doi: 10.1186/s12967-023-04581-x (PMC10583486; doi:10.1186/s12967-023-04581-x)
Supplement: Supplementary file 2 — Additional file 2. Supplementary Table S1–5. [file 12967_2023_4581_MOESM2_ESM.docx]

**Table S1 The primers for overexpression vector construction and siRNA sequence**

| Name | sequence（5’-3’） | |
| --- | --- | --- |
| LINC02913 | Forward | ctagcgtttaaacttaagcttAGGCAAAGCCGGGAGAAA |
|  | Reverse | tgctggatatctgcagaattcCCTCACCCACCTTTCCTTTGC |
| HIF1A | Forward | ctagcgtttaaacttaagcttATGGAGGGCGCCGGCGGC |
|  | Reverse | tgctggatatctgcagaattcTCAGTTAACTTGATCCAAAGCTCTG |
| IGF1R | Forward | ctagcgtttaaacttaagcttATGAAGTCTGGCTCCGGAGG |
|  | Reverse | tgctggatatctgcagaattcTCAGCAGGTCGAAGACTGGG |
| Si-NC |  | UUCUCCGAACGUGUCACGUTT |
|  |  | ACGUGACACGUUCGGAGAATT |
| Si-HIF1A |  | GCUAUUCACCAAAGUUGAATT |
|  |  | UUCAACUUUGGUGAAUAGCTT |

**Table S2 The primers for qRT-PCR**

| Name |  | sequence（5’-3’） |
| --- | --- | --- |
| LINC02913 | Forward | CGGGAGAAACTGCTGAGACGA |
|  | Reverse | TGAAGGGAAGGGCTCGGAT |
| IGF1R | Forward | ATGCTGACCTCTGTTACCTCT |
|  | Reverse | GGCTTATTCCCCACAATGTAGTT |
| α-tubulin | Forward | GGAGGGGATGGAGGAAGGTG |
|  | Reverse | ACACCAACCTCCTCATAATCCTTCT |

**Table S4 The primers for luciferase reporter vector construction**

| Name |  | sequence（5’-3’） |
| --- | --- | --- |
| psicheck2-proLINC02913-WT | Forward | aattctaggcgatcgctcgagACCCGTGCGTGGCCCCCA |
|  | Reverse | attttattgcggccagcggccgcCCCCCTCTAGTTGCAAAAAAAA |
| psicheck2-proLINC02913-MUT | Forward | GCGGCaaatgtacctTGTTCCCCGCTTTCGGGC |
| Mutant aaatgtacct | Reverse | ACAaggtacatttGCCGCCCCCGAGCTGCGGAC |

**Table S5 The predicted LINC02913 interacted proteins by RNAact (https://rnact.crg.eu/)(Prediction Score >20).**

| RNA |  | Protein |  |  |  | Prediction (catRAPID) | |
| --- | --- | --- | --- | --- | --- | --- | --- |
| Transcript Symbol | Ensembl Transcript ID | Gene | UniProt Accession | Length | Protein Status | Prediction Score | Prediction z-Score |
| C9orf106-201 | ENST00000316786 | NISCH | Q9Y2I1 | 1504 aa |  | 29.03 | ■■■□□ 2.24 |
| C9orf106-201 | ENST00000316786 | CCDC180 | Q9P1Z9 | 1646 aa |  | 25.02 | ■■□□□ 1.6 |
| C9orf106-201 | ENST00000316786 | ZCCHC6 | Q5VYS8 | 1495 aa | Known RBP | 24.41 | ■■□□□ 1.5 |
| C9orf106-201 | ENST00000316786 | DCAF8L2 | P0C7V8 | 631 aa |  | 24.27 | ■■□□□ 1.48 |
| C9orf106-201 | ENST00000316786 | SHROOM4 | Q9ULL8 | 1493 aa |  | 24.22 | ■■□□□ 1.47 |
| C9orf106-201 | ENST00000316786 | DCAF1 | Q9Y4B6 | 1507 aa |  | 23.82 | ■■□□□ 1.4 |
| C9orf106-201 | ENST00000316786 | ABCC9 | O60706 | 1549 aa |  | 23.79 | ■■□□□ 1.4 |
| C9orf106-201 | ENST00000316786 | SUPT5H | O00267 | 1087 aa | Known RBP | 23.41 | ■■□□□ 1.34 |
| C9orf106-201 | ENST00000316786 | MYO15B | Q96JP2 | 1530 aa |  | 23.4 | ■■□□□ 1.34 |
| C9orf106-201 | ENST00000316786 | SCRIB | Q14160 | 1630 aa |  | 23.33 | ■■□□□ 1.32 |
| C9orf106-201 | ENST00000316786 | NACAD | O15069 | 1562 aa |  | 23.3 | ■■□□□ 1.32 |
| C9orf106-201 | ENST00000316786 | RB1CC1 | Q8TDY2 | 1594 aa |  | 23.08 | ■■□□□ 1.29 |
| C9orf106-201 | ENST00000316786 | UNC13A | Q9UPW8 | 1703 aa |  | 22.72 | ■■□□□ 1.23 |
| C9orf106-201 | ENST00000316786 | CECR2 | Q9BXF3 | 1484 aa |  | 22.69 | ■■□□□ 1.22 |
| C9orf106-201 | ENST00000316786 | RSF1 | Q96T23 | 1441 aa | Predicted RBP | 22.69 | ■■□□□ 1.22 |
| C9orf106-201 | ENST00000316786 | PABPN1 | Q86U42 | 306 aa | Known RBP eCLIP | 22.59 | ■■□□□ 1.21 |
| C9orf106-201 | ENST00000316786 | MROH2B | Q7Z745 | 1585 aa |  | 22.53 | ■■□□□ 1.2 |
| C9orf106-201 | ENST00000316786 | BICRA | Q9NZM4 | 1560 aa |  | 22.47 | ■■□□□ 1.19 |
| C9orf106-201 | ENST00000316786 | CHIC1 | Q5VXU3 | 224 aa |  | 22.4 | ■■□□□ 1.18 |
| C9orf106-201 | ENST00000316786 | WIZ | O95785 | 1651 aa |  | 22.37 | ■■□□□ 1.17 |
| C9orf106-201 | ENST00000316786 | BAZ1A | Q9NRL2 | 1556 aa | Predicted RBP | 22.35 | ■■□□□ 1.17 |
| C9orf106-201 | ENST00000316786 | SMARCA4 | P51532 | 1647 aa |  | 22.26 | ■■□□□ 1.15 |
| C9orf106-201 | ENST00000316786 | PELP1 | Q8IZL8 | 1130 aa | Known RBP | 22.18 | ■■□□□ 1.14 |
| C9orf106-201 | ENST00000316786 | TOP2A | P11388 | 1531 aa | Known RBP | 22.01 | ■■□□□ 1.11 |
| C9orf106-201 | ENST00000316786 | CCDC88B | A6NC98 | 1476 aa |  | 21.9 | ■■□□□ 1.1 |
| C9orf106-201 | ENST00000316786 | SMARCA2 | P51531 | 1590 aa |  | 21.87 | ■■□□□ 1.09 |
| C9orf106-201 | ENST00000316786 | PEG3 | Q9GZU2 | 1588 aa |  | 21.86 | ■■□□□ 1.09 |
| C9orf106-201 | ENST00000316786 | TRIM41 | Q8WV44 | 630 aa |  | 21.86 | ■■□□□ 1.09 |
| C9orf106-201 | ENST00000316786 | ATAD2B | Q9ULI0 | 1458 aa |  | 21.86 | ■■□□□ 1.09 |
| C9orf106-201 | ENST00000316786 | EIF4G1 | Q04637 | 1599 aa | Known RBP | 21.78 | ■■□□□ 1.08 |
| C9orf106-201 | ENST00000316786 | BAZ1B | Q9UIG0 | 1483 aa | Known RBP | 21.76 | ■■□□□ 1.07 |
| C9orf106-201 | ENST00000316786 | MYT1 | Q01538 | 1121 aa |  | 21.72 | ■■□□□ 1.07 |
| C9orf106-201 | ENST00000316786 | LOC105371045 | A0A1W2PR82 | 267 aa |  | 21.71 | ■■□□□ 1.07 |
| C9orf106-201 | ENST00000316786 | FAM9A | Q8IZU1 | 332 aa | Predicted RBP | 21.66 | ■■□□□ 1.06 |
| C9orf106-201 | ENST00000316786 | ABCC8 | Q09428 | 1581 aa |  | 21.62 | ■■□□□ 1.05 |
| C9orf106-201 | ENST00000316786 | NCAPD3 | P42695 | 1498 aa |  | 21.56 | ■■□□□ 1.04 |
| C9orf106-201 | ENST00000316786 | HMGXB3 | Q12766 | 1538 aa |  | 21.5 | ■■□□□ 1.03 |
| C9orf106-201 | ENST00000316786 | EHMT2 | Q96KQ7 | 1210 aa |  | 21.5 | ■■□□□ 1.03 |
| C9orf106-201 | ENST00000316786 | CC2D2A | Q9P2K1 | 1620 aa |  | 21.42 | ■■□□□ 1.02 |
| C9orf106-201 | ENST00000316786 | WRN | Q14191 | 1432 aa | Predicted RBP eCLIP | 21.34 | ■■□□□ 1.01 |
| C9orf106-201 | ENST00000316786 | SOGA1 | O94964 | 1423 aa |  | 21.27 | ■■□□□ 1 |
| C9orf106-201 | ENST00000316786 | SYNJ1 | O43426 | 1573 aa |  | 21.26 | ■□□□□ 0.99 |
| C9orf106-201 | ENST00000316786 | RUSC2 | Q8N2Y8 | 1516 aa |  | 21.2 | ■□□□□ 0.98 |
| C9orf106-201 | ENST00000316786 | CFTR | P13569 | 1480 aa |  | 21.19 | ■□□□□ 0.98 |
| C9orf106-201 | ENST00000316786 | DNAJC5B | Q9UF47 | 199 aa |  | 21.1 | ■□□□□ 0.97 |
| C9orf106-201 | ENST00000316786 | CADPS | Q9ULU8 | 1353 aa |  | 21.1 | ■□□□□ 0.97 |
| C9orf106-201 | ENST00000316786 | CSRNP3 | Q8WYN3 | 585 aa |  | 21.07 | ■□□□□ 0.96 |
| C9orf106-201 | ENST00000316786 | TOP2B | Q02880 | 1626 aa |  | 21.06 | ■□□□□ 0.96 |
| C9orf106-201 | ENST00000316786 | CUX1 | P39880 | 1505 aa |  | 21.02 | ■□□□□ 0.96 |
| C9orf106-201 | ENST00000316786 | EEA1 | Q15075 | 1411 aa |  | 21.01 | ■□□□□ 0.95 |
| C9orf106-201 | ENST00000316786 | BIVM-ERCC5 | R4GMW8 | 1640 aa |  | 20.99 | ■□□□□ 0.95 |
| C9orf106-201 | ENST00000316786 | NES | P48681 | 1621 aa |  | 20.98 | ■□□□□ 0.95 |
| C9orf106-201 | ENST00000316786 | PCGF6 | Q9BYE7 | 350 aa |  | 20.98 | ■□□□□ 0.95 |
| C9orf106-201 | ENST00000316786 | PRDM2 | Q13029 | 1718 aa |  | 20.97 | ■□□□□ 0.95 |
| C9orf106-201 | ENST00000316786 | UBTF | P17480 | 764 aa | Known RBP | 20.93 | ■□□□□ 0.94 |
| C9orf106-201 | ENST00000316786 | FANCD2 | Q9BXW9 | 1451 aa |  | 20.89 | ■□□□□ 0.93 |
| C9orf106-201 | ENST00000316786 | GOLGA3 | Q08378 | 1498 aa |  | 20.84 | ■□□□□ 0.93 |
| C9orf106-201 | ENST00000316786 | TNIK | Q9UKE5 | 1360 aa |  | 20.82 | ■□□□□ 0.92 |
| C9orf106-201 | ENST00000316786 | CEP162 | Q5TB80 | 1403 aa |  | 20.82 | ■□□□□ 0.92 |
| C9orf106-201 | ENST00000316786 | CEP164 | Q9UPV0 | 1460 aa |  | 20.8 | ■□□□□ 0.92 |
| C9orf106-201 | ENST00000316786 | HRC | P23327 | 699 aa |  | 20.8 | ■□□□□ 0.92 |
| C9orf106-201 | ENST00000316786 | PPARGC1B | Q86YN6 | 1023 aa | Known RBP | 20.8 | ■□□□□ 0.92 |
| C9orf106-201 | ENST00000316786 | RAPGEF2 | Q9Y4G8 | 1499 aa |  | 20.79 | ■□□□□ 0.92 |
| C9orf106-201 | ENST00000316786 | KIF21B | O75037 | 1637 aa |  | 20.77 | ■□□□□ 0.92 |
| C9orf106-201 | ENST00000316786 | FGD5 | Q6ZNL6 | 1462 aa |  | 20.77 | ■□□□□ 0.91 |
| C9orf106-201 | ENST00000316786 | LAMC3 | Q9Y6N6 | 1575 aa |  | 20.76 | ■□□□□ 0.91 |
| C9orf106-201 | ENST00000316786 | KIF27 | Q86VH2 | 1401 aa |  | 20.74 | ■□□□□ 0.91 |
| C9orf106-201 | ENST00000316786 | RANGAP1 | P46060 | 587 aa | Predicted RBP | 20.72 | ■□□□□ 0.91 |
| C9orf106-201 | ENST00000316786 | TRIM26 | Q12899 | 539 aa | Predicted RBP | 20.72 | ■□□□□ 0.91 |
| C9orf106-201 | ENST00000316786 | CUX2 | O14529 | 1486 aa |  | 20.7 | ■□□□□ 0.9 |
| C9orf106-201 | ENST00000316786 | TTBK1 | Q5TCY1 | 1321 aa | Predicted RBP | 20.7 | ■□□□□ 0.9 |
| C9orf106-201 | ENST00000316786 | TOPBP1 | Q92547 | 1522 aa |  | 20.68 | ■□□□□ 0.9 |
| C9orf106-201 | ENST00000316786 | DNMBP | Q6XZF7 | 1577 aa |  | 20.66 | ■□□□□ 0.9 |
| C9orf106-201 | ENST00000316786 | PRX | Q9BXM0 | 1461 aa |  | 20.65 | ■□□□□ 0.9 |
| C9orf106-201 | ENST00000316786 | RAD54L2 | Q9Y4B4 | 1467 aa |  | 20.65 | ■□□□□ 0.9 |
| C9orf106-201 | ENST00000316786 | PDS5B | Q9NTI5 | 1447 aa |  | 20.64 | ■□□□□ 0.9 |
| C9orf106-201 | ENST00000316786 | PRKCSH | P14314 | 528 aa | Predicted RBP | 20.63 | ■□□□□ 0.89 |
| C9orf106-201 | ENST00000316786 | URB2 | Q14146 | 1524 aa | Known RBP | 20.63 | ■□□□□ 0.89 |
| C9orf106-201 | ENST00000316786 | POLA1 | P09884 | 1462 aa | Predicted RBP | 20.62 | ■□□□□ 0.89 |
| C9orf106-201 | ENST00000316786 | KIF21A | Q7Z4S6 | 1674 aa |  | 20.62 | ■□□□□ 0.89 |
| C9orf106-201 | ENST00000316786 | CLIP1 | P30622 | 1438 aa |  | 20.59 | ■□□□□ 0.89 |
| C9orf106-201 | ENST00000316786 | **IGF1R** | P08069 | 1367 aa |  | 20.58 | ■□□□□ 0.89 |
| C9orf106-201 | ENST00000316786 | ERICH3 | Q5RHP9 | 1530 aa |  | 20.57 | ■□□□□ 0.88 |
| C9orf106-201 | ENST00000316786 | SCAF1 | Q9H7N4 | 1312 aa | Known RBP | 20.55 | ■□□□□ 0.88 |
| C9orf106-201 | ENST00000316786 | ABCA10 | Q8WWZ4 | 1543 aa |  | 20.5 | ■□□□□ 0.87 |
| C9orf106-201 | ENST00000316786 | CARMIL1 | Q5VZK9 | 1371 aa |  | 20.49 | ■□□□□ 0.87 |
| C9orf106-201 | ENST00000316786 | TDRD9 | Q8NDG6 | 1382 aa | Known RBP | 20.45 | ■□□□□ 0.86 |
| C9orf106-201 | ENST00000316786 | VPS8 | Q8N3P4 | 1428 aa |  | 20.4 | ■□□□□ 0.86 |
| C9orf106-201 | ENST00000316786 | WDR97 | A6NE52 | 1622 aa |  | 20.38 | ■□□□□ 0.85 |
| C9orf106-201 | ENST00000316786 | MRC2 | Q9UBG0 | 1479 aa |  | 20.37 | ■□□□□ 0.85 |
| C9orf106-201 | ENST00000316786 | ARAP1 | Q96P48 | 1450 aa |  | 20.34 | ■□□□□ 0.85 |
| C9orf106-201 | ENST00000316786 | GAPVD1 | Q14C86 | 1478 aa |  | 20.32 | ■□□□□ 0.84 |
| C9orf106-201 | ENST00000316786 | BCL11A | Q9H165 | 835 aa |  | 20.31 | ■□□□□ 0.84 |
| C9orf106-201 | ENST00000316786 | TARBP1 | Q13395 | 1621 aa | Known RBP | 20.31 | ■□□□□ 0.84 |
| C9orf106-201 | ENST00000316786 | ERCC6 | Q03468 | 1493 aa |  | 20.31 | ■□□□□ 0.84 |
| C9orf106-201 | ENST00000316786 | ABCA5 | Q8WWZ7 | 1642 aa |  | 20.31 | ■□□□□ 0.84 |
| C9orf106-201 | ENST00000316786 | ARHGAP35 | Q9NRY4 | 1499 aa |  | 20.3 | ■□□□□ 0.84 |
| C9orf106-201 | ENST00000316786 | APLP2 | Q06481 | 763 aa |  | 20.28 | ■□□□□ 0.84 |
| C9orf106-201 | ENST00000316786 | AQR | O60306 | 1485 aa | Known RBP eCLIP | 20.26 | ■□□□□ 0.83 |
| C9orf106-201 | ENST00000316786 | CUL7 | Q14999 | 1698 aa |  | 20.25 | ■□□□□ 0.83 |
| C9orf106-201 | ENST00000316786 | P3H3 | Q8IVL6 | 736 aa |  | 20.23 | ■□□□□ 0.83 |
| C9orf106-201 | ENST00000316786 | WDR62 | O43379 | 1518 aa |  | 20.22 | ■□□□□ 0.83 |
| C9orf106-201 | ENST00000316786 | ZNF608 | Q9ULD9 | 1512 aa | Predicted RBP | 20.19 | ■□□□□ 0.82 |
| C9orf106-201 | ENST00000316786 | KANK4 | Q5T7N3 | 995 aa | Predicted RBP | 20.15 | ■□□□□ 0.82 |
| C9orf106-201 | ENST00000316786 | IFT140 | Q96RY7 | 1462 aa |  | 20.13 | ■□□□□ 0.81 |
| C9orf106-201 | ENST00000316786 | THOC2 | Q8NI27 | 1593 aa | Known RBP | 20.13 | ■□□□□ 0.81 |
| C9orf106-201 | ENST00000316786 | PBRM1 | Q86U86 | 1689 aa |  | 20.13 | ■□□□□ 0.81 |
| C9orf106-201 | ENST00000316786 | CTTNBP2 | Q8WZ74 | 1663 aa |  | 20.11 | ■□□□□ 0.81 |
| C9orf106-201 | ENST00000316786 | FHOD3 | Q2V2M9 | 1422 aa |  | 20.1 | ■□□□□ 0.81 |
| C9orf106-201 | ENST00000316786 | HNRNPUL2-BSCL2 | H3BQZ7 | 746 aa | Predicted RBP | 20.08 | ■□□□□ 0.81 |
| C9orf106-201 | ENST00000316786 | GRIN2B | Q13224 | 1484 aa |  | 20.07 | ■□□□□ 0.8 |
| C9orf106-201 | ENST00000316786 | ERICH6 | Q7L0X2 | 663 aa |  | 20.07 | ■□□□□ 0.8 |
| C9orf106-201 | ENST00000316786 | ATP1B4 | Q9UN42 | 357 aa | Predicted RBP | 20.06 | ■□□□□ 0.8 |
| C9orf106-201 | ENST00000316786 | FHAD1 | B1AJZ9 | 1412 aa |  | 20.05 | ■□□□□ 0.8 |
| C9orf106-201 | ENST00000316786 | ADAMTS12 | P58397 | 1594 aa |  | 20.04 | ■□□□□ 0.8 |
| C9orf106-201 | ENST00000316786 | HNRNPUL2 | Q1KMD3 | 747 aa | Known RBP | 20.03 | ■□□□□ 0.8 |
| C9orf106-201 | ENST00000316786 | EIF4G3 | O43432 | 1585 aa | Known RBP | 20.03 | ■□□□□ 0.8 |
